# Role of pre-operative red cell distribution width estimation in the prediction of in-hospital mortality after off-pump coronary artery bypass grafting

**DOI:** 10.1186/s13019-021-01612-w

**Published:** 2021-08-13

**Authors:** Dharmendra Joshi, Md. Abir Tazim Chowdhury, Md. Alauddin, Redoy Ranjan, Omar Sadeque Khan, Md. Rezwanul Hoque

**Affiliations:** Department of Cardiac Surgery, Bangabandhu Sheikh Mujib Medical University, Dhaka, Bangladesh

**Keywords:** Red cell distribution width (RDW), Off-pump coronary artery bypass, Risk factors, Predictors, Mortality

## Abstract

**Background:**

Red cell distribution width (RDW) level is routinely provided in a simple and inexpensive complete blood count report. However, RDW is sometimes overlooked. Recently a higher RDW level is found associated with postoperative mortality after off-pump coronary artery bypass. Many risk-prediction tools are available, like the European System for Cardiac Operative Risk Evaluation, Society of Thoracic Surgeons score, etc. but all need improvement for better prediction. So, a new risk-factor should be discovered which is simple enough for clinical use and cost-effective, and improves the risk assessment tools that help to predict and avoid preventable mortality following cardiac surgery.

**Methods:**

The prospective study was conducted, taking a total of 150 patients of coronary artery disease who underwent elective isolated off-pump coronary artery bypass. The study population was grouped according to their preoperative RDW level as Group A (RDW ≤ 14%), Group B (RDW 14–16%), and Group C (RDW ≥ 16%). The receiver operating characteristic (ROC) curve was constructed and multivariate regression analysis was done to see the predictive value of RDW for in-hospital mortality.

**Results:**

The mortality rate was 2.7%, N = 150. ROC curve revealed Area Under the Curve 0.841 and *p* = 0.020 that indicates the RDW as the reliable predictor for in-hospital mortality. Multivariate regression analysis showed the RDW to be the only variable independently predicting in-hospital mortality after off-pump coronary artery bypass among possible haematological predictors. (OR 1.838, 95% CI 1.061–3.186, *p* = 0.030).

**Conclusion:**

Preoperative raised RDW level is a novel predictor of in-hospital mortality after off-pump coronary artery bypass. Further studies should be done to determine the associated mechanism.

**Supplementary Information:**

The online version contains supplementary material available at 10.1186/s13019-021-01612-w.

## Introduction

Various risks assessment methods are used to evaluate the risk of mortality after cardiac surgery but even broadly utilized risk models, like the European System for Cardiac Operative Risk Evaluation (EuroSCORE) have some confinements, including poor performance in the elderly and overestimated mortality, especially among isolated coronary artery bypass grafting patients. Along these lines, new risk factors need to be discovered which ought to be simple enough for clinical use and cost-effective [1].

In the last decade, some of the haematological parameters including red cell distribution width (RDW) is currently showing some genuine promise to demonstrate a measure of the general inflammatory activity with prognostic information related to ischemic disorders and the pathogenesis of the cerebrovascular disease [2]. Raised RDW has shown potential use in preoperative risk stratification of patients to identify post-operative mortality but this is not studied thoroughly yet [3]. Although the exact mechanisms are still not elucidated clearly, RDW is being considered as a global marker of oxidative stress, chronic inflammation, and cardiovascular disease risk [4].

Our objectives were to estimate the preoperative RDW level in patients who undergo off-pump coronary artery bypass (OPCAB) and evaluate its role for the prediction of in-hospital mortality after OPCAB. However, the study did not aim to find the causes of mortality or prove raised RDW as the cause of in-hospital mortality after OPCAB as it remains equivocal regarding the mechanism between raised RDW level and mortality.

## Materials and methods

### Patient selection

**Fig. 1:**
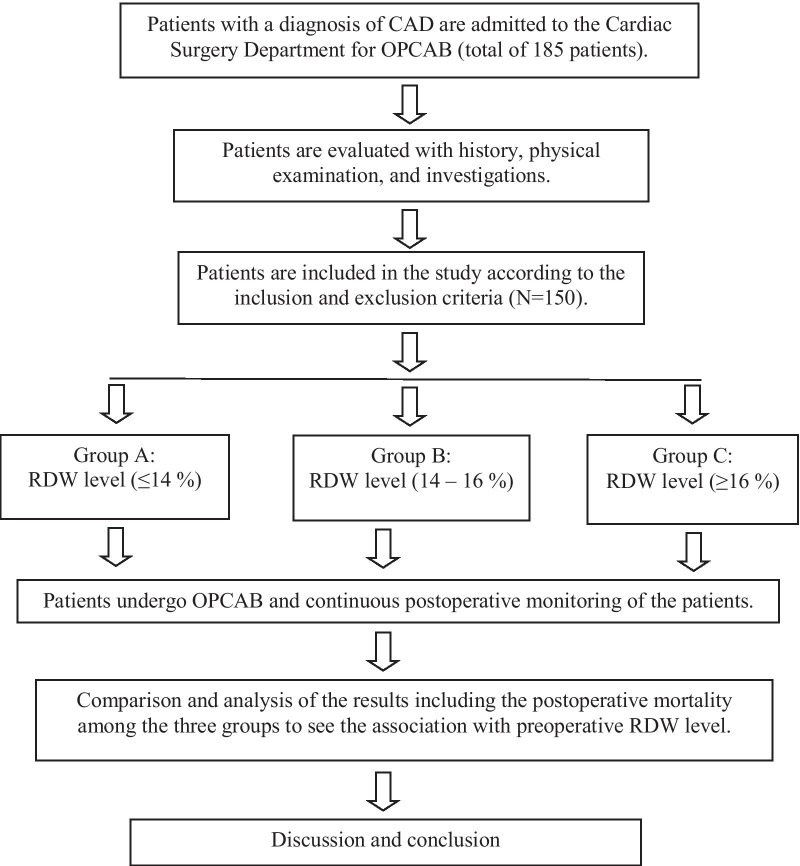
Flow chart of the study design. CAD: Coronary artery disease, OPCAB: Off-pump coronary artery bypass, RDW: Red cell distribution width

This prospective study was conducted by grouping a total of 150 patients, who underwent elective isolated OPCAB between July 2017 to August 2019, into three groups according to the preoperative RDW level viz. Group A (RDW ≤ 14%, n = 75), Group B (RDW 14–16%, n = 36), and Group C (RDW ≥ 16%, n = 39). The detail of the study design is provided in Fig. 1. The ethical clearance from the Institutional Review Board (IRB reference no. 2018/8107) and informed written consent from every patient under the study were taken.

### Exclusion criteria

Patients with coronary artery disease having: (1) previous treatment for anaemia and preoperative blood transfusion ≤ 3 months before surgery, (2) clinical evidence of active infection 7 days before surgery, (3) concomitant valve surgery along with CABG, (4) congestive cardiac failure, atrial fibrillation, thyroid disorder, chronic renal or hepatic disease and overt/active hematologic proliferative diseases at the time of preoperative evaluation, (5) preoperative need for respiratory support, inotropic drug support or intra-aortic balloon pump (IABP), and (6) operation on an emergency basis.

### Operational definitions

#### RDW

It is the quotient of a standard deviation of red blood cell volume and its mean volume. A higher RDW value means more heterogeneity in erythrocytes volume [5]. It is routinely reported along with the automated complete blood count (CBC) [6]. The term ‘width’ does not refer to the width of the red blood cell, rather the width of the volume curve [7]. RDW is recorded statistically as RDW-SD (Standard Deviation) and RDW-CV (Coefficient of variation), depending on the kinds of hemocytometer used [8]. We have taken only RDW-CV in this study for analysis which is indicated as only “RDW”, whenever applied.

#### In-hospital mortality/death

Nashef et al*.* defined it as any of the following: (1) death in the same hospital where the operation was done, before discharge from the hospital; (2) death in the same hospital or at another hospital but before discharge from hospital [9].

### Laboratory

With all aseptic precaution, about 2 ml of whole blood was collected preoperatively by venipuncture of the patient. Complete Blood Count including RDW level was measured using Hematology Autoanalyzer Sysmex XN-2000. The normal reference range for RDW in our laboratory was 11.6–14.0%.

### Statistical analysis

Statistical analysis was conducted by Statistical Package for Social Science (SPSS) version 23.0 for windows software. Categorical data were shown as frequency and percentage. Continuous data were shown as mean ± standard deviation. Comparisons between groups were made with the Chi-Square test and Fisher’s exact test for categorical data whereas One-Way ANOVA for continuous parametric (normally distributed) data and Kruskal–Wallis test for continuous non-parametric data. The statistical measurement of the effect of RDW on in-hospital mortality was assessed by constructing a receiver operating characteristic (ROC) curve. Univariate regression analysis followed by Multivariate regression analysis was conducted to identify the independent association of preoperative RDW level with in-hospital mortality. Observations were recorded as statistically significant if a p-value ≤ 0.05. The statistical analysis was performed by the professional statistician.

## Results

### Socio-demographic parameters

The baseline characteristics of the study population are presented in Table 1. The mortality rate was 2.7% (N = 150) among all the study populations. It is interesting to observe that three mortality occurred in Group C, one in Group B, and none in Group A, which was statistically significant (*p* = 0.037). All clinical history, preoperative diagnoses, and risk factors were also analyzed and were found to be statistically not significant (*p* > 0.05). The comparison of EuroSCORE II between groups was found statistically significant (*p* = 0.001).

**Table 1:** Baseline characteristics of the patients compared according to RDW level

Variables	Group A	Group B	Group C	*p* value
**Age (years)**	54.51 ± 9.33	53.75 ± 7.67	56.64 ± 9.30	0.334
**Gender (male)**	65 (86.7)	32 (88.9)	35 (89.7)	0.947
**BMI (kg/m**^**2**^**)**	24.02 ± 3.32	23.13 ± 2.30	23.84 ± 3.28	0.359
**Risk factors**				
Smoker	49 (65.3)	27 (75.0)	29 (74.4)	0.481
IHD/MI	57 (76.0)	29 (80.6)	31 (75.5)	0.872
PVD	1 (1.3)	0 (0.0)	3 (7.7)	0.120
Hypertension	53 (70.7)	20 (61.1)	19 (48.7)	0.070
Diabetes mellitus	32 (42.7)	18 (50.0)	20 (51.3)	0.650
**EuroSCORE II**	0.94 ± 0.33	1.05 ± 0.44	1.31 ± 0.75	**0.001**
**Preoperative diagnoses**				
SVD	2 (2.7)	0 (0.0)	0 (0.0)	
DVD	11 (14.7)	4 (11.1)	4 (10.3)	
TVD	36 (48.0)	24 (66.7)	26 (66.7)	0.475
SVD with LMD	1 (1.3)	1 (2.8)	1 (2.6)	
DVD with LMD	7 (9.3)	0 (0.0)	2 (5.1)	
TVD with LMD	18 (24.0)	7 (19.4)	6 (15.4)	

Data are shown as Mean ± SD. Figure within parenthesis indicates percentage. *p* value < 0.05 is considered statistically significant

*BMI* Body Mass Index, *IHD* ischemic heart disease, *MI* Myocardial Infarction, *PVD* peripheral vessel disease, *EuroSCORE* European System for Cardiac Operative Risk Evaluation, *SVD* single vessel disease, *DVD* double vessel disease, *TVD* triple vessel disease, *LMD* left main vessel disease

### Preoperative investigations and operative parameters

Analyses of preoperative investigations were done (Table 2). Only Mean corpuscular volume (MCV), Mean corpuscular haemoglobin (MCH), Mean corpuscular haemoglobin concentration (MCHC), mean platelet volume (MPV), and Platelet Distribution Width (PDW) were found statistically significant (*p* = 0.014, *p* =  < 0.001, *p* = 0.009, *p* = 0.030 and *p* =  < 0.001 respectively). Left ventricular ejection fraction (LVEF) was normal in most of the study populations, two patients had LVEF < 30%, each in Group B and Group C, and LVEF 30- 50% in 37.3% patients (Group A), 44.4% patients (Group B), and 23.1% patients (Group C). Triple vessel disease (TVD) was the most frequent preoperative diagnosis among the three groups (Table 1). The mean number of distal anastomoses performed was 2.79 ± 0.58, 3.00 ± 0.63, and 3.18 ± 0.60 among Group A, Group B, and Group C patients respectively (*p* = 0.004) (Table 2).

**Table 2:** Preoperative routine investigations and operative data compared according to RDW level

Variables	Group A	Group B	Group C	*p* value
**Complete Blood Count**				
Haemoglobin (gm/dL)	12.69 ± 1.54	12.60 ± 1.26	12.41 ± 1.45	0.612
ESR (mm in 1st hour)	28.28 ± 16.91	30.11 ± 21.36	28.44 ± 17.68	0.878
Platelet count (× 10^12^/L)	274.88 ± 71.59	261.86 ± 81.52	246.64 ± 78.09	0.162
Total WBC count (× 10^9^/L)	8.49 ± 2.25	8.54 ± 1.74	8.30 ± 2.07	0.866
Neutrophil/lymphocyte ratio	2.12 ± 0.95	1.98 ± 0.79	2.12 ± 0.73	0.724
**Absolute Indices**				
PCV (%)	37.28 ± 4.19	37.68 ± 3.99	38.37 ± 3.98	0.402
MCV (fL)	85.65 ± 5.02	83.18 ± 7.48	82.06 ± 7.90	**0.014**
MCH (pg)	29.10 ± 2.63	27.65 ± 2.24	26.67 ± 3.52	** < 0.001**
MCHC (pg)	33.80 ± 3.56	33.23 ± 1.49	32.13 ± 1.06	**0.009**
MPV (fL)	10.00 ± 1.19	9.73 ± 1.61	9.32 ± 1.04	**0.030**
PDW (fL)	12.69 ± 2.68	13.87 ± 2.72	16.23 ± 2.64	** < 0.001**
**C-Reactive Protein (mg/L)**	3.54 ± 4.06	5.24 ± 6.19	2.83 ± 3.02	0.082
**RBS (mmol/L)**	7.11 ± 2.61	8.47 ± 3.92	8.83 ± 4.22	0.340
**Echocardiography**				
LVEF (%)	54.54 ± 10.11	51.75 ± 10.33	56.33 ± 10.41	0.153
RWMA	60 (80.0)	27 (75.0)	36 (92.3)	0.116
Left Atrial size (mm)	33.31 ± 3.92	33.83 ± 4.67	34.10 ± 4.59	0.612
**Operative data**				
** Number of distal anastomoses**	2.79 ± 0.58	3.00 ± 0.63	3.18 ± 0.60	**0.004**
** Name of conduits used**				
Internal mammary artery (left)	74 (98.7)	36 (100.0)	38 (97.4)	0.999
Saphenous venous graft	73 (97.3)	36 (100.0)	39 (100.0)	0.738
Radial artery	0 (0.0)	0 (0.0)	1 (2.6)	0.500

Data are shown as Mean ± SD. Figure within parenthesis indicates percentage. *p* value < 0.05 is considered statistically significant

*ESR* erythrocyte sedimentation rate, *WBC* white blood cell, *PCV* packed cell volume, *MCV* mean corpuscular volume, *MCH* mean corpuscular haemoglobin, *MCHC* mean corpuscular haemoglobin concentration, *MPV* mean platelet volume, *PDW* platelet distribution width, *RBS* random blood sugar, *LVEF* left ventricular ejection fraction, *RWMA* regional wall motion abnormality

### ROC (receiver operating characteristic) curve

**Fig. 2:**
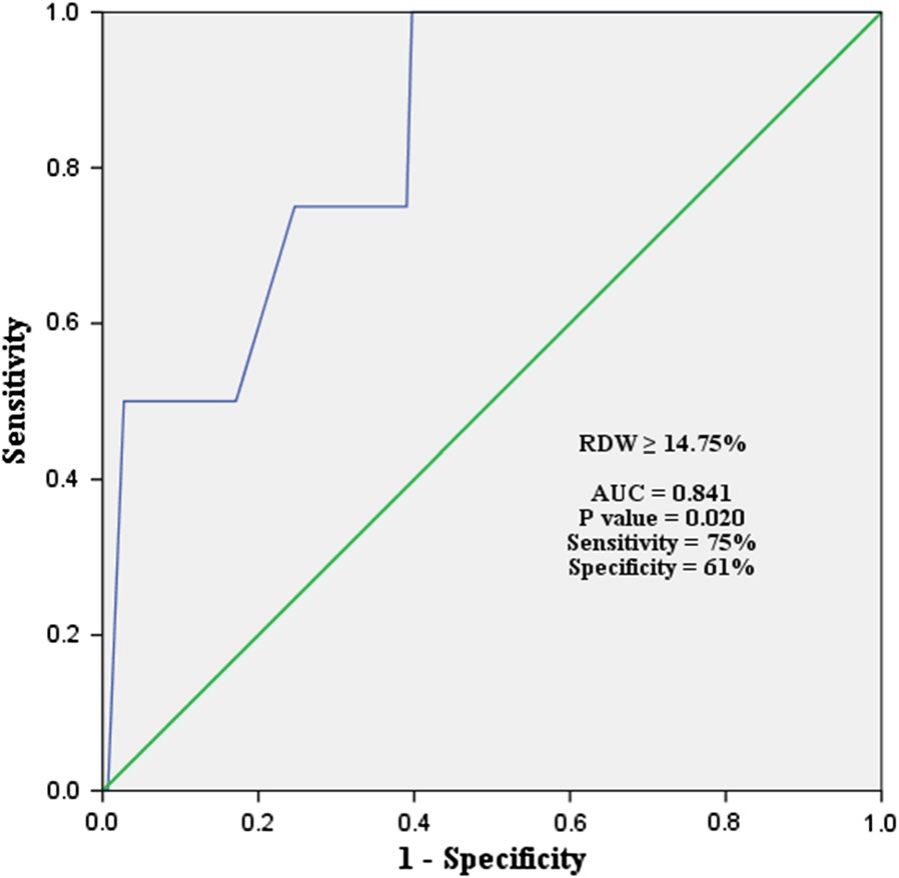
Receiving operating characteristic (ROC) curve of RDW for the prediction of mortality after off-pump coronary artery bypass grafting. RDW: red cell distribution width, AUC: area under curve

A ROC curve was constructed to determine the accuracy of the predictive value of preoperative RDW level for in-hospital mortality (Fig. 2). The area under the curve (AUC) was 0.841 with a sensitivity of 75% and specificity of 61% at RDW ≥ 14.75% (95% CI 0.682–0.999, *p* = 0.020).

### Univariate and multivariate regression analysis

Univariate regression analysis was performed for all the statistically significant variables of the study and most of the known predictors of mortality after OPCAB like age, gender, history of smoking, hypertension, diabetes mellitus, WBC count, Neutrophil/Lymphocyte ratio, haemoglobin, CRP, and Platelet count, etc. Only RDW with OR 1.568, 95% CI 1.076–2.284, *p* = 0.019, and EuroSCORE II with OR 5.809, 95% CI 1.778- 18.979, *p* = 0.004 were found significant. Then, the relationship between baseline haematological predictors and in-hospital postoperative mortality was assessed by the use of multivariate regression analysis. The well-known haematological predictors of the mortality were also forced into the multivariable model (Table 3). Among the variables, the preoperative plasma RDW level was found to be the only independent predictor (OR 1.838, 95% CI 1.061–3.186, *p* = 0.030).

**Table 3:** Tomographic variables examined in the multivariate regression analysis

Variables	OR	95.0% CI (OR)	p value
RDW	1.838	1.061–3.186	**0.030**
Haemoglobin	1.931	0.315–11.853	0.477
PCV	0.724	0.346–1.511	0.389
WBC	1.637	0.830–3.229	0.155
N/L ratio	0.578	0.083–4.021	0.580
Platelet	0.980	0.959–1.002	0.081
MPV	0.863	0.255–2.915	0.812
PDW	1.083	0.677–4.37	0.740

*p* value < 0.05 is considered statistically significant

*RDW* red cell distribution width, *PCV* packed cell volume, *WBC* White blood cell, *N/L* neutrophil/lymphocyte, *MPV* mean platelet volume, *PDW* platelet distribution width, *CI* confidence interval, *OR* odds ratio

### Supplementary data

A comparison and analysis of remaining routine investigations are provided in Additional file 1: Table S1. Similarly, preoperative coronary angiogram and spirometry findings are also presented in Additional file 1: Table S2 and Table S3 respectively. All results showed *p* > 0.05 (see Supplementary material).

## Discussion

RDW is primarily used for differential diagnosis of anaemia. Increased RDW values are considered as an indicator of anaemia due to nutritional deficiencies like iron, folate, vitamin B12 deficiencies. RDW is also considered as a novel biomarker that mirrors numerous physiological impedances identified with atherosclerosis and CAD. Recently, the predictive role of RDW for some cardiovascular, pulmonary, and renal disorders has been studied. Although the mechanisms underlying raised RDW remain unclear, many hypotheses have been proposed like oxidative stress, inflammation, and cytokines release that may reduce the iron metabolism which decreases the life span of RBC and increases RDW [2, 6, 10]. The relationship between chronic inflammation and increase RDW is also advocated by a study in which RDW corresponded with both the erythrocyte sedimentation rate and C-reactive protein (CRP) [11].

In this study, out of the 150 patients, the male was predominant in all three groups. Male occupied 88% of the total study population which reflects the male predominance for CAD [12]. Many studies have shown the significant impact of age, female gender, smoking, peripheral vascular disease, hypertension, diabetes mellitus, ischemic heart disease/myocardial infarction, and other risk factors on in-hospital mortality of patients [7, 13] but these were well controlled before the operation in this study. Hence, the results were not statistically significant in this study.

Preoperative routine investigations of the patients were done to fulfil the inclusion criteria like the absence of anaemia, good glycemic control, normal renal function, liver function, thyroid function, etc. Only MCV, MCH, MCHC, MPV, and PDW were found statistically significant [11]. Low LVEF and large left atrial (LA) size are considered prognostic factors in the postoperative adverse outcome after cardiac surgery [14]. However, LA size and LVEF were mostly within the normal range in this study population (two patients had LVEF < 30%), which may be due to extensive exclusion criteria put in patient selection. [1, 15]. The number of distal anastomoses done during OPCAB had a significant impact on in-hospital mortality among the groups. The number of distal anastomoses required was comparatively more in group C (RDW ≥ 16%). The study conducted by Gurbuz et al. showed similar results [16]. The intracoronary shunt was used for all the patients during anastomosis for better coronary blood flow. The patency of the conduits placed was confirmed during operation by its easy and unobstructed blood flow. However, the conduits were not assessed in the postoperative period by other means like a coronary angiogram.

Our university is a tertiary referral centre and many cases are operated with limited resources, infrastructure, and experience. Hence, a little higher mortality of 2.7% was found in this study. However, the mortality rate is improving each year with the advancement in skill, experience, and support systems like coronary angiogram, transesophageal echocardiography (TEE), sustained low-efficiency dialysis (SLED), etc. EuroSCORE II is considered a reliable cardiac operative mortality predictor tool. Most of the studies including our study found the comparative difference in EuroSCORE between groups [9]. The study found an independent relationship between higher RDW levels and the possibility of postoperative in-hospital mortality. The receiver operating curve (ROC) also showed Area Under Curve (AUC) 0.841 with a sensitivity of 75% and specificity of 61% at RDW ≥ 14.75% which proved the predictive value of the RDW level for in-hospital mortality (*p* = 0.020). The scale of AUC ranges from 0.5–1.0. The closure AUC is to 1.00 the better its accuracy [17]. Multivariate regression analysis showed plasma RDW level was the only independent predictor of postoperative in-hospital mortality among other haematological variables. This result corresponded to the findings of Katlandur et al. where RDW was found independent predictor of mortality (OR 1.860, 95% CI 1.105–3.132, *p* = 0.020) [2].

In this study postoperative MI, atrial fibrillation, ventricular tachycardia, ventricular fibrillation, excessive bleeding, renal failure, etc. were found more in patients with increased RDW. The data regarding these morbidities and possible causes of mortality are not discussed in detail in this study because it is beyond the scope/objectives of the study and it remains ambiguous regarding the mechanism between increased RDW and mortality. Few researchers advocate that systemic inflammation and impaired renal function play a pivotal role in the underlying pathological processes [18]. It is also believed that perioperative MI results in an inflammatory process which is indicated by raised plasma RDW level, and the association of perioperative myocardial injury and adverse clinical outcomes is well-proven [3]. Further studies are required to confirm the mechanism between raised RDW level and mortality after OPCAB.

## Study limitations

This prospective study was limited by a small sample size because we considered the study cohort exclusive for patients undergoing elective isolated OPCAB with extensive inclusion/exclusion criteria for a limited period. The study was done in a single centre. So, the sample represents only a small portion of patients undergoing OPCAB. The study focused only on the mortality, rather than the morbidities because it did not aim to prove raised RDW as the cause of mortality or find the causes of mortality after OPCAB. However, the study was conducted to evaluate preoperative raised RDW level as the predictor of postoperative in-hospital mortality after OPCAB.

## Conclusion

The preoperative raised plasma RDW level is a predictor of in-hospital mortality after elective OPCAB. So, an inexpensive plasma RDW measurement should be incorporated in the risk prediction tools for even better prediction of mortality after OPCAB. This will ultimately encourage cardiac surgeons to be more vigilant for high-risk patients and prevent avoidable mortality after cardiac surgery. Multi centre-based, larger prospective studies (including the high risk and on-pump CABG cases) are needed to validate the findings of the study.

## Supplementary Information

**Additional file 1: Supplemental Table 1**. Remaining preoperative routine investigations.** Supplemental Table 2**. Preoperative Coronary angiogram findings.** Supplemental Table 3**. Preoperative Spirometry findings.

## Data Availability

Yes.

## Abbreviations

CAD: Coronary artery disease
CBC: Complete blood count
EDTA: Ethylenediaminetetraacetic acid
EuroSCORE: European System for Cardiac Operative Risk Evaluation
LVEF: Left ventricular ejection fraction
MCH: Mean corpuscular haemoglobin
MCHC: Mean corpuscular haemoglobin concentration
MCV: Mean corpuscular volume
MPV: Mean platelet volume (MPV)
OPCAB: Off-pump coronary artery bypass
PDW: Platelet distribution width
RDW: Red cell distribution width
ROC: Receiver operating characteristic
