# Supplementary material for: Role of pre-operative red cell distribution width estimation in the prediction of in-hospital mortality after off-pump coronary artery bypass grafting
Source: J Cardiothorac Surg. 2021 Aug 13;16:232. doi: 10.1186/s13019-021-01612-w (PMC8361624; doi:10.1186/s13019-021-01612-w)
Supplement: Supplementary file 1 — Additional file 1: Supplemental Table 1. Remaining preoperative routine investigations. Supplemental Table 2. Preoperative Coronary angiogram findings. Supplemental Table 3. Preoperative Spirometry findings. [file 13019_2021_1612_MOESM1_ESM.docx]

**SUPPLEMENTAL MATERIALS:**

**Supplemental Table 1: Remaining preoperative routine investigations**

**Table S1: Remaining preoperative routine investigations compared according to RDW level**

| **Preoperative investigations** | **Group A** | **Group B** | **Group C** | **p value** |
| --- | --- | --- | --- | --- |
| FBS (mmol/L) | 7.55 ± 2.61 | 8.14 ± 2.29 | 8.05 ± 2.40 | 0.620 |
| HbA1c (%) | 7.02 ± 1.54 | 7.85 ± 1.98 | 7.23 ± 1.66 | 0.172 |
| Serum Creatinine (mg/dL) | 1.09 ± 0.21 | 1.07 ± 0.24 | 1.12 ± 0.25 | 0.595 |
| Serum TSH (mlU/L) | 1.93 ± 1.17 | 1.70 ± 1.17 | 2.06 ± 1.16 | 0.405 |
| **Lipid Profile:** |  |  |  |  |
| Total Cholesterol (mg/dL) | 150.81 ± 41.01 | 156.42 ± 37.55 | 167.19 ± 57.82 | 0.349 |
| HDL (mg/dL) | 35.16 ± 7.03 | 41.64 ± 22.77 | 36.32 ± 7.88 | 0.261 |
| LDL (mg/dL) | 84.38 ± 34.03 | 85.73 ± 37.00 | 85.62 ± 39.52 | 0.986 |
| Triglyceride (mg/dL) | 178.96 ± 96.15 | 182.37 ± 91.56 | 190.19 ± 122.20 | 0.908 |
| **Liver Function Tests:** |  |  |  |  |
| Total Bilirubin (mg/dL) | 0.59 ± 0.24 | 0.53 ± 0.24 | 0.57 ± 0.29 | 0.502 |
| SGPT (U/L) | 35.63 ± 21.83 | 37.14 ± 39.57 | 35.07 ± 26.63 | 0.947 |
| Serum Albumin (gm/L) | 41.70 ± 6.69 | 39.96 ± 4.67 | 42.16 ± 4.57 | 0.211 |

FBS: Fasting blood sugar, HbA1c: Hemoglobin A1c, TSH: Thyroid-stimulating hormone, HDL: High-density lipoprotein, LDL: Low-density lipoprotein, SGPT: Serum glutamic-pyruvic transaminase. Data are shown as Mean ± SD.

**Supplemental Table 2: Preoperative Coronary angiogram findings**

**Table S2: Preoperative Coronary angiogram findings compared according to RDW level**

| **CAG: Artery involvement** | **Group A** | **Group B** | **Group C** | **p value** |
| --- | --- | --- | --- | --- |
| **LMCA involvement** |  |  |  |  |
| Normal | 46 (61.3) | 27 (75.0) | 28 (71.8) |  |
| Group: I (< 50% stenosis) | 16 (21.3) | 4 (11.1) | 6 (15.4) | 0.823 |
| Group: II (50%-70% stenosis) | 6 (8.0) | 2 (5.6) | 3 (7.7) |  |
| Group: III (> 70% stenosis) | 7 (9.3) | 3 (8.3) | 2 (5.1) |  |
| **LAD involvement** |  |  |  |  |
| Normal | 0 (0.0) | 0 (0.0) | 0 (0.0) |  |
| Group: I (< 50% stenosis) | 3 (4.0) | 3 (8.3) | 6 (15.4) | 0.192 |
| Group: II (50%-70% stenosis) | 11 (14.7) | 4 (11.1) | 2 (5.1) |  |
| Group: III (> 70% stenosis) | 61 (81.3) | 29 (80.6) | 31 (79.5) |  |
| **LCX involvement** |  |  |  |  |
| Normal | 11 (14.7) | 7 (19.4) | 2 (5.1) |  |
| Group: I (< 50% stenosis) | 8 (10.7) | 4 (11.1) | 1 (2.6) | 0.700 |
| Group: II (50%-70% stenosis) | 6 (8.0) | 5 (13.9) | 6 (15.4) |  |
| Group: III (> 70% stenosis) | 50 (66.7) | 20 (55.6) | 30 (76.9) |  |
| **RCA involvement** |  |  |  |  |
| Normal | 8 (10.7) | 2 (5.6) | 3 (7.7) |  |
| Group: I (< 50% stenosis) | 11 (14.7) | 4 (11.1) | 3 (7.7) | 0.835 |
| Group: II (50%-70% stenosis) | 2 (2.7) | 1 (2.8) | 0 (0.0) |  |
| Group: III (> 70% stenosis) | 54 (72.0) | 29 (80.6) | 33 (88.6) |  |

CAG: Coronary angiogram, LMCA: Left main coronary artery, LAD: Left anterior descending artery, LCX: Left circumflex artery, RCA: Right coronary artery. Figure within parenthesis indicates percentage.

**Supplemental Table 3: Preoperative Spirometry findings**

**Table S3: Preoperative Spirometry findings compared according to RDW level**

| **Spirometry findings** | **Group A** | **Group B** | **Group C** | **p value** |
| --- | --- | --- | --- | --- |
| Normal | 39 (52.0) | 19 (52.8) | 23 (59.0) |  |
| Mild Restriction | 5 (6.7) | 6 (16.7) | 6 (15.4) |  |
| Moderate Restriction | 0 (0.0) | 2 (5.6) | 0 (0.0) |  |
| Mild Obstruction | 6 (8.0) | 0 (0.0) | 2 (5.1) | 0.136 |
| Moderate Obstruction | 1 (1.3) | 2 (5.6) | 1 (2.6) |  |
| Mixed Disease | 1 (1.3) | 0 (0.0) | 0 (0.0) |  |
| ^*^Not Applicable | 23 (30.7) | 7 (19.4) | 7 (17.9) |  |

*Left main disease. Figure within parenthesis indicates percentage.
